# Predicting Social Anxiety From Global Positioning System Traces of College Students: Feasibility Study

**DOI:** 10.2196/10101

**Published:** 2018-07-04

**Authors:** Mehdi Boukhechba, Philip Chow, Karl Fua, Bethany A Teachman, Laura E Barnes

**Affiliations:** ^1^ Systems and Information Engineering Department School of Engineering and Applied Science University of Virginia Charlottesville, VA United States; ^2^ Department of Psychology University of Virginia Charlottesville, VA United States

**Keywords:** mental health, mHealth, mobility, GPS, social anxiety disorder

## Abstract

**Background:**

Social anxiety is highly prevalent among college students. Current methodologies for detecting symptoms are based on client self-report in traditional clinical settings. Self-report is subject to recall bias, while visiting a clinic requires a high level of motivation. Assessment methods that use passively collected data hold promise for detecting social anxiety symptoms and supplementing self-report measures. Continuously collected location data may provide a fine-grained and ecologically valid way to assess social anxiety in situ.

**Objective:**

The objective of our study was to examine the feasibility of leveraging noninvasive mobile sensing technology to passively assess college students’ social anxiety levels. Specifically, we explored the different relationships between mobility and social anxiety to build a predictive model that assessed social anxiety from passively generated Global Positioning System (GPS) data.

**Methods:**

We recruited 228 undergraduate participants from a Southeast American university. Social anxiety symptoms were assessed using self-report instruments at a baseline laboratory session. An app installed on participants’ personal mobile phones passively sensed data from the GPS sensor for 2 weeks. The proposed framework supports longitudinal, dynamic tracking of college students to evaluate the relationship between their social anxiety and movement patterns in the college campus environment. We first extracted the following mobility features: (1) cumulative staying time at each different location, (2) the distribution of visits over time, (3) the entropy of locations, and (4) the frequency of transitions between locations. Next, we studied the correlation between these features and participants’ social anxiety scores to enhance the understanding of how students’ social anxiety levels are associated with their mobility. Finally, we used a neural network-based prediction method to predict social anxiety symptoms from the extracted daily mobility features.

**Results:**

Several mobility features correlated with social anxiety levels. Location entropy was negatively associated with social anxiety (during weekdays, *r*=−0.67; and during weekends, *r*=−0.51). More (vs less) socially anxious students were found to avoid public areas and engage in less leisure activities during evenings and weekends, choosing instead to spend more time at home after school (4 pm-12 am). Our prediction method based on extracted mobility features from GPS trajectories successfully classified participants as high or low socially anxious with an accuracy of 85% and predicted their social anxiety score (on a scale of 0-80) with a root-mean-square error of 7.06.

**Conclusions:**

Results indicate that extracting and analyzing mobility features may help to reveal how social anxiety symptoms manifest in the daily lives of college students. Given the ubiquity of mobile phones in our society, understanding how to leverage passively sensed data has strong potential to address the growing needs for mental health monitoring and treatment.

## Introduction

Social anxiety is marked by an extreme fear of being scrutinized and judged by others in social or performance situations [1]. In addition to being a widespread problem among college students, a high social anxiety level is associated with a low quality of life. For example, socially anxious individuals suffer from impaired academic functioning and relationships [2]. The American College Health Association reported that 40% of students reported feeling “overwhelming anxiety” at least once in the preceding year [3].

Current techniques to identify social anxiety are typically based on self-report via questionnaires or interviews in traditional clinical settings, where only small numbers of people can be monitored and client motivation is required to seek an assessment. This approach is inadequate and fails to meet the growing needs of mental health monitoring and treatment on college campuses. As a result, many individuals who should seek help never receive any. For example, according to the Anxiety and Depression Association of America, 36% people with social anxiety disorder report having symptoms for 10 or more years before seeking help [1].

Mental health symptoms can be indirectly assessed through both subjective (eg, self-report surveys and interviews) and objective (physiological variables such as heart rate) methods. Such methods have largely been employed in clinical or laboratory settings, which limits the ecological validity of findings. To increase the generalizability of findings, researchers have tried to understand mental health through noninvasive and real-time data collected from people’s everyday lives. For example, studies using surveys to repeatedly sample people’s momentary affective experiences over time have found that individuals with high (vs low) social anxiety symptoms report more anger and fewer and less intense positive emotions [4,5]. While studies that administer repeated surveys offer a glimpse of the socioemotional aspects of daily life, regularly prompting individuals to answer questions also raises the issue of participation burden.

Importantly, embedded mobile phone sensors (eg, accelerometers, light sensors, Global Positioning System [GPS]) are now advanced enough to allow for passive and continuous data collection [6,7] and are increasingly being used to enhance the understanding of the relationship between objective behavior and mental health status, such as bipolar disorder [8], anxiety and depression [7,9-13], and Alzheimer disease and dementia [14]. Digital phenotyping, which is a term used for describing this new approach of measuring behavior from mobile phones and wearable sensors, is already revealing new aspects of behaviors that appear clinically relevant [15]. For example, Saeb et al [16,17] provided preliminary evidence that extracting location-based mobility features could be used to detect depression and anxiety levels. Barnet et al [18] were able to use passively generated mobile phone data to identify statistically significant anomalies in patients with schizophrenic behavior in the days prior to relapse. The above studies show the importance of analyzing behaviors to better understand mental states.

Because social anxiety is marked by intense fear of social scrutiny, passively sensed location information may reveal key markers that can be used to detect a high distress level. Semantic locations (ie, the type of social location someone visits) might be particularly important in the context of social anxiety. For example, individuals with social anxiety might systematically avoid specific places, such as those of leisure, or choose to spend peak social hours by isolating themselves at home. Thus, analyzing GPS data from college students and the types of places they frequent might provide crucial information about key mobility features associated with social anxiety levels. Some examples of mobility features include how long students spend at different types of locations (eg, home, leisure) and how often they frequent those locations.

Important contributions [7,9,12,17,19] have been made to determine how passively sensed mobile phone data relates to users’ mental health statuses and stress levels. We followed the key steps from these valuable early studies and extended the scope of features and questions addressed, as outlined below: (1) recruiting participants and deploying a mobile app for data collection; (2) collecting data such as GPS locations, recognized activities, or self-reported data from participants during the study; (3) assessing participants’ mental health status using validated clinical measures; (4) extracting meaningful features or metrics from participants’ data (eg, time spent at each different location); and (5) correlating these features or metrics with participants’ mental health status (eg, Pearson correlation between number of distinct locations and clinically validated measures).

This study builds on prior work in several ways and improves our understanding of the relationships between social anxiety symptoms and daily routines. In this paper, we introduce and analyze a set of passively extracted spatiotemporal features that enhance our understanding of the temporal and spatial dynamics of behavioral patterns (eg, regularly visiting a location during specific hours) of socially anxious students, which may allow for more precise, personalized interventions. We also propose a hierarchical social anxiety prediction method based on neural networks. This work may ultimately help researchers and clinicians to passively and remotely monitor patients’ social anxiety levels. The primary aim of this paper was not to test specific hypotheses, but rather to explore a framework for using passively collected GPS data to detect social anxiety levels.

## Methods

### Study Organization and Data Collection

Participants were undergraduate students with varying social anxiety levels, recruited from undergraduate psychology classes that provide course credit as a participation incentive. Because some participants met or exceeded their course credit limit, a subset of participants was eligible to receive a small amount of monetary compensation (up to a maximum of US $40). Students were recruited through email advertisements as well as through an undergraduate study participant pool. The decision to recruit university students was based on two reasons: (1) there are high social anxiety levels among young adults, and (2) recruiting young adults in a university setting provides a relatively homogeneous sample in terms of life phase, psychological stressors, and life experiences, thereby eliminating a wide variety of potential confounding factors.

After receiving approval from the university Institutional Review Board, 228 participants were recruited. Social anxiety level was first assessed via the Social Interaction Anxiety Scale (SIAS) [20] in a prestudy screening battery offered to select undergraduate psychology classes in exchange for course credit. The SIAS contains 20 items, each rated from 0 to 4. Generally, a higher SIAS score (specifically, higher than 34) [21] indicates a higher risk of having social anxiety concerns; a low score indicates a lower risk for social anxiety concerns.

**Figure 1:**
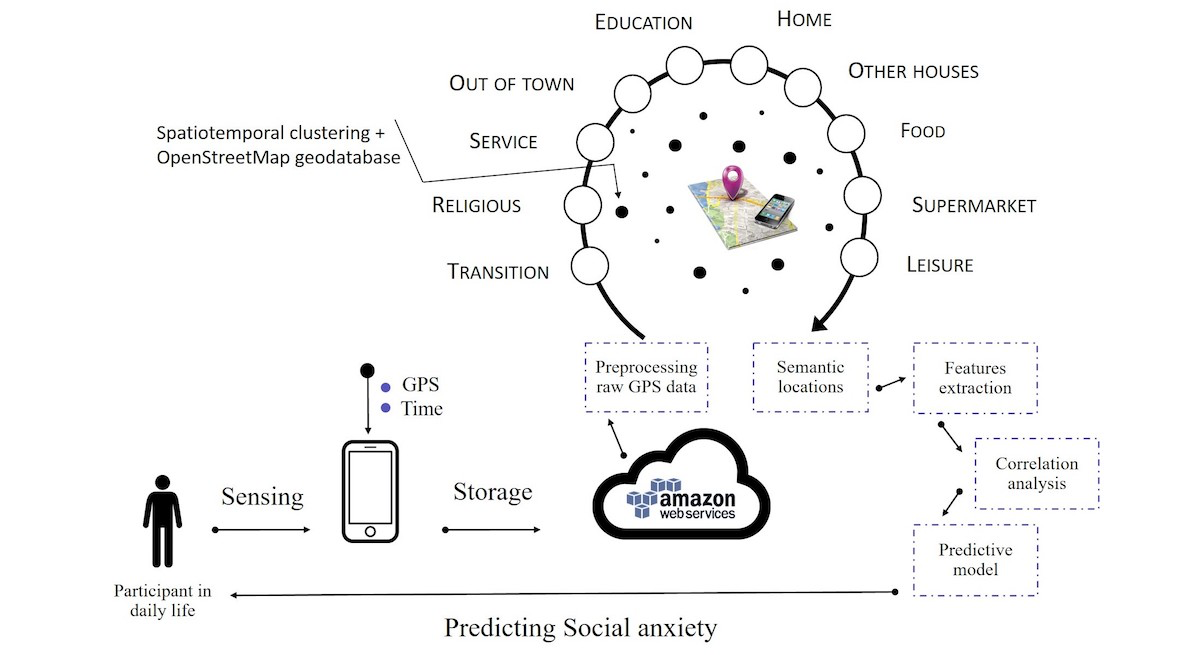
Social anxiety monitoring framework.

Following informed consent, a custom mobile app (Sensus) [22] was installed on participants’ personal mobile phone (either IOS or Android device). As shown in Figure 1, participants were informed that the app passively collected the GPS location information every 150 seconds and uploaded it to an Amazon Web Services S3 server. After the 2-week experiment was completed, researchers could access all participants’ raw GPS data for analysis.

### Global Positioning System Data Preprocessing

**Figure 2:**
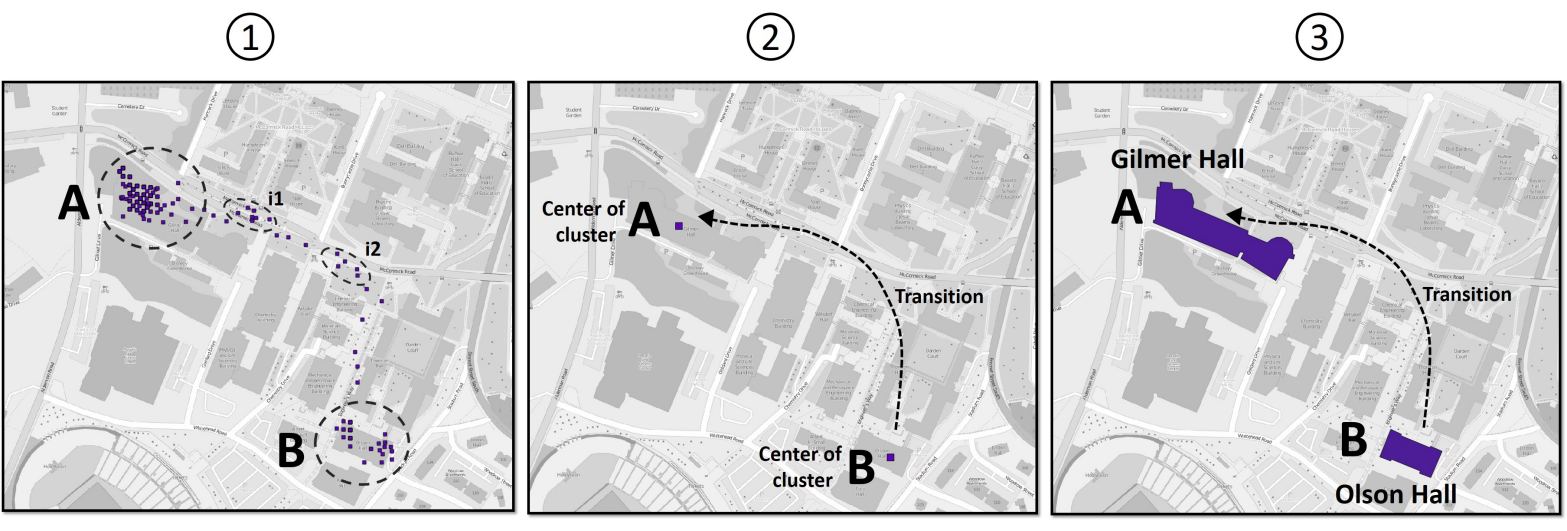
Illustration of our time-based clustering algorithm using real GPS data retrieved from one participant in the study. In (1), GPS locations are clustered using the algorithm described in Textbox 1. In (2), the trajectory is summarized to two places (A and B) and the transition state, which aggregates all GPS points between the clusters A and B. Finally, in (3), the clusters A and B are labeled using OSM data. GPS: Global Positioning System, OSM: open street map.

Participants’ raw GPS data were parsed by semantic locations (eg, restaurant, campus area, and shops) by combining a spatiotemporal clustering algorithm and OpenStreetMap (OSM) geodatabase [23]. Specifically, we first clustered participants’ GPS locations using time and space dimensions, and then, each cluster was associated with a semantic location using OSM data [24] (Figure 2).

Our clustering algorithm is inspired by the work of Kang et al [25], and it aims to eliminate the intermediate locations between important places and determine the number of clusters (important places) autonomously. The core idea guiding our approach is to cluster the locations along the time axis. As a new location measurement is read, the new location is compared with previous locations. If the new location is moving away from previous locations (within a certain distance of each other—a parameter of our algorithm), the new location is considered to belong to a different cluster than the one for the previous locations. Otherwise, it is considered to belong to the previous cluster. If a cluster’s time duration is longer than the threshold (the second parameter of our algorithm), the cluster is considered to be a significant place (see A and B in Figure 2); otherwise, it is ignored (see i1 and i2 in Figure 2).

The algorithm is depicted in Textbox 1 (*d* and *t* are our distance and time threshold parameters). When a new location measurement event is generated, the cluster function is invoked. The current cluster *cl* is the set of location measurements that belong to the current cluster. The pending location *pl* is used to eliminate outliers. Even if the new location is far away from the current cluster (distance is larger than the distance threshold d), the algorithm does not start a new cluster right away with the new location. Instead, the algorithm waits for the next location to determine if the user is really moving away from the cluster or if the location reading was just a spurious outlier. The *Places* contain the significant places where the user stays longer than the time threshold *t*.

**Spatiotemporal clustering algorithm.**

**Input**: measured location *loc*

**Output**: current cluster *cl*, pending location *pl*, significant places *Places*

**if** distance (*cl*, *loc*) < d **then**

add *loc* to *cl*

*pl* = null

**else**

**if** *pl* ≠ null **then**

**if** duration(*cl*) > *t* then

**if** contain long gaps(*cl*) **then**

remove gaps from *cl*

**end if**

add *cl* to *Places*

**end if**

clear *cl*

add *pl* to *cl*

**if** distance (*cl, pl*) < *d* **then**

add *loc* to *cl*

*pl* = null

**else**

*pl* = *loc*

**end if**

**else**

*pl* = *loc*

**end if**

**end if**

Our algorithm was tuned using d=60 m and t=600 seconds. These values appeared to give the best clustering results for our data; they have also been reported in the literature to give the best performance for spatiotemporal clustering algorithms [25].

After detecting the significant clusters, we transformed each GPS cluster to a meaningful semantic label using OSM data. Each GPS cluster’s centroid is associated to a geographic entity (eg, in Figure 2, cluster A is associated to Gilmer Hall and cluster B to Olsson Hall, both of which are buildings on the university campus) using a spatial query in our geodatabase powered by OSM. The semantic data obtained from OSM is then classified to one of the following classes:

- **Home**: our algorithm has been trained to recognize “Home” as the place having a house OSM tag (eg, apartment, dormitory, house, etc) where a subject stays the most between 10 pm and 9 am; see [23] for more details about OSM tags.
- **Other houses**: all houses other than “Home”; in this study, given all participants are college students, other houses were assumed to mostly be friends’ houses.
- **Education**: eg, university and libraries
- **Leisure**: eg, sports locations, pubs, cinemas, and coffee shops
- **Food**: eg, dining halls and restaurants including fast food joints
- **Supermarket**: all full-service grocery stores
- **Religious**: all places of worship, including churches, mosques, cathedrals, synagogues, temples, etc
- **Service**: eg, bank, post office, courthouse
- **Out of town**: locations outside of the city where the study was conducted
- **In transition**: going from one place to another

Note that ideally, food places would be merged with leisure and supermarkets with service classes. However, we decided to separate them because we discovered a particular pattern that high socially anxious participants (SAP) share in terms of time spent at food places and supermarkets, which will be discussed in the next section.

When constructing GPS clusters labeled with semantics, we verified if the users’ data contained GPS gaps. We defined a GPS gap *g*_i_ as a minimum 10-minute time span where GPS data were missing. Gaps could be caused by different events, such as turning the phone off or “killing” the app. For gaps ∈ [5 min, 30 min], we compared the cluster *cl*_i_ and the cluster *cl*_i+1_, created before and after the gap, respectively. If the 2 clusters had the same semantic labels, we considered that the user did not change his or her location during *g*_i_; thus, we merged the clusters *cl*_i_*, cl*_i+1_, and the gap *g*_i_. However, if the 2 clusters had different semantic labels, we assigned the “Transition” label to *g*_i_; ie, the user changed locations during this gap.

For gaps exceeding 30 minutes in duration, we removed the corresponding time periods from the experiment (see Textbox 1, line 7), because it was hard to predict what the participants did during such long gaps.

### Mobility Feature Extraction

After detecting participants’ visited places and labeling them using OSM, we used the semantic labels to identify the following mobility features for each participant:

**Cumulative Staying Time** in each type of location: Given a type of location and a specific participant, this metric characterized the percentage of total time that the participant spent at one type of location during a specific time window (eg, during a day, during mornings vs afternoons).

**Distribution of visits** for each type of location: Given a type of location and a specific participant, this metric calculated the density distribution of time of visits over the time of day. For instance, if a participant was more likely to go to leisure places during evenings, we would find more density during the evening periods for this type of locations. We introduced this metric because cumulative staying time captures only time spent at each different location without recording when these visits happened; for instance, spending 2 hours at the university during mornings was different than that during evenings.

**Location entropy**: This metric was calculated using the entropy of Shannon [26] to measure how each participant’s time was distributed over different location classes, where *p_i_* is the percentage of time spent at location *i* and *n* is the total number of visited locations:

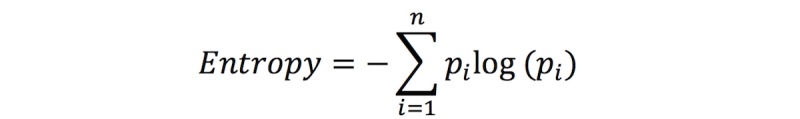



**Transition Frequency** from one type of location to another: Given a specific participant and two types of locations (eg, “Home” and “Work”), this metric characterized the frequency at which the participant transited from one type of location to another. This metric was applied unidirectionally; for example, the transition frequency of “Home” ⟶ “Work” and the transition frequency of “Work” ⟶ “Home” were different.

## Results

### Participants

**Figure 3:**
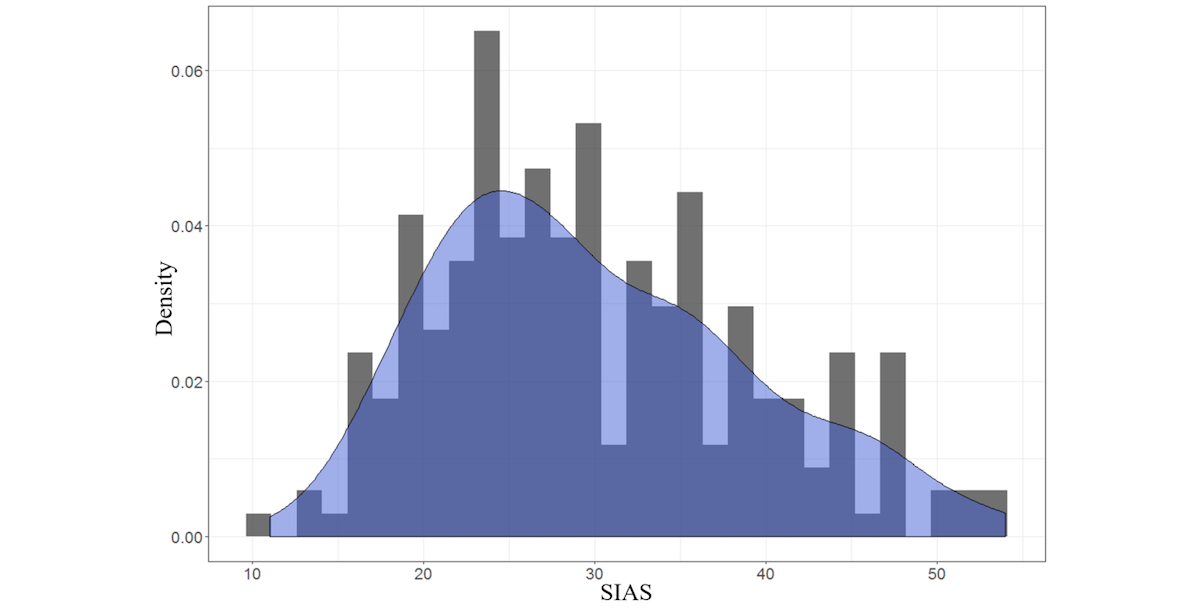
The distribution of Social Interaction Anxiety Scale (SIAS) scores for recruited participants. The Epanechnikov kernel function was used to compute the density estimates presented in this figure.

Participants comprised 228 university students (mean age 19.43 [SD 2.92] years; 141/228, 62%, females). Participants reported their race or ethnicity as white (118/228, 52%), Asian (61/228, 27%), black (12/228, 5%), Latino (5/228, 2%), and multiracial (32/228, 14%). Figure 3 shows the distribution of SIAS scores among the 228 participants. The SIAS scores ranged between 11 and 54 with a mean score 29.91 (SD 9.1).

The goal of our study was: (1) to understand the relationship between the extracted mobility features and the preassessed SIAS score and (2) to investigate whether the extracted features could predict SIAS scores. Consequently, in the next two sections, we will first analyze the relationship between the mobility features and social anxiety and then investigate whether these features can accurately predict social anxiety.

### Mobility Data Analysis

In this section, we present the results of our analysis investigating the relationship between social anxiety levels (using the preassessed SIAS measures) and the four extracted mobility metrics: cumulative staying time, distribution of visits, location entropy, and transition frequency.

#### Cumulative Staying Time

We calculated the Pearson correlation between each participant’s average daily cumulative staying time at each different location and his or her SIAS score to identify the direction (positive or negative) and strength of each correlation. To assess the reliability of the correlations, we also calculated significance levels (*P* value).

**Figure 4:**
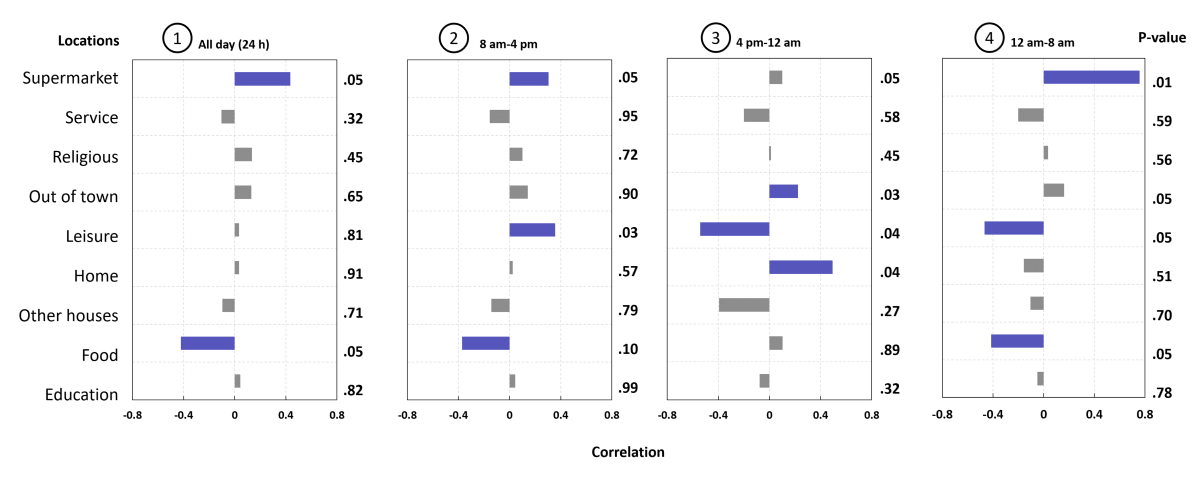
The correlations between time spent at each different type of location and Social Interaction Anxiety Scale (SIAS) scores using different time windows. In (1), we have presented the correlations on a daily basis, while in the other figures, we have focused on specific portions of the day, ie, between 8 am and 4 pm, between 4 pm and 12 am, and between 12 am and 8 am. The x-axis represents the correlation significance; the left y-axis describes the type of locations, and the right y-axis represents the P value of the Pearson correlation in that specific type of location. Correlations having a coefficient *r*>0.2 and a strength *P*<0.05 are presented in purple.

Results presented in graph 1 of Figure 4 show that daily time spent at some locations was associated with the SIAS score. Indeed, time spent at food locations, such as restaurants and dining halls, was negatively correlated with the SIAS score. However, time spent at supermarkets was positively correlated with the SIAS score. This suggests that high SAP are more likely to buy food from supermarkets so they can eat at home, perhaps to avoid social interactions at restaurants.

College students may have common mobility patterns that bias the daily correlation analysis, such as class times at the university following a typical schedule. In order to find the hidden patterns between the cumulative staying time and SIAS score, we analyzed correlations in different daily time epochs: 8 am-4 pm, 4 pm-12 am, and 12 am-8 am. Results presented in graphs 2, 3, and 4 of Figure 4 suggest the following:

- Similar to the 24-hour analysis (Figure 4, graph 1), the time spent at food locations was negatively correlated with the SIAS score, while time spent at supermarkets was positively correlated with the SIAS score in both the 12 am-8 am and 8 am-4 pm time windows.
- Time spent doing leisure activities was positively correlated with the SIAS score between 8 am and 4 pm, while the rest of time it was negatively correlated with the SIAS score. This suggests that high SAP prefer to do their leisure activities between 8 am and 4 pm, rather than during the evenings. This may reflect the social demands typical of different types of leisure activities done during the day versus evening (eg, it is more normative to be alone at a coffee shop than at a pub or bar).
- We did not find a correlation between time spent at home and the SIAS score between 12 am and 4 pm (Figure 4, graphs 2 and 4), which may simply indicate that no matter how socially anxious students were, they tended to stay at home (sleeping) between 12 am-8 am and leave home to go to school between 8 am and 4pm. However, during the time after typical school hours (between 4 pm and 12 am, when students have the choice to stay at home or not), we found a positive correlation between the SIAS score and time spent at home (Figure 4, graph 3). This finding is consistent with a prior work that associates time spent at home with depressive and social isolation symptoms [27].
- Finally, we found a small correlation (0.22) between time spent out of town and the SIAS score during the 4 pm-12 am time window, perhaps indicating that more socially anxious students leave the university to visit familiar individuals or family, rather than engaging in campus night life, which may have more demands to be social with unfamiliar individuals.

After analyzing the correlation between cumulative staying time and the SIAS score, we studied the difference between cumulative staying time during weekdays versus weekends for high (SIAS score ≥ 34) versus low (SIAS < 34) SAP. A score of 34 is an established clinical cutoff for the SIAS score to classify a subject as high or low socially anxious [21]. The reason for this analysis is that students’ patterns may differ between weekdays and weekends. For instance, maybe the time spent at the university is not a good predictor of social anxiety during weekdays, but it is during weekends when students presumably have more autonomy in determining their schedule (because classes are not set).

**Table 1:** The difference between high and low socially anxious students in terms of average daily time spent (in minutes) at each different location during weekdays versus weekends.

Location	Weekdays		Weekends	
	High^a^	Low^a^	High^a^	Low^a^
Supermarket	6.64^b^	1.41^b^	16.64^b^	10.41^b^
Service	6.97	1.83	9.97	5.73
Religious	7.35	1.63	10.35	2.75
Out of town	38.25^b^	22.33^b^	88.25^b^	27.8^b^
Leisure	26.04^b^	45.19^b^	43.16^b^	75.09^b^
Home	434.74	386.94	594.74^b^	469.02^b^
Other houses	17.63^b^	24.81^b^	16.21^b^	64.81^b^
Food	3.78^b^	12.97^b^	9.78^b^	32.97^b^
Education	338.44	316.88	148.79^b^	102.98^b^

^a^Social anxiety levels were classified to high and low using SIAS score=34 as cutoff.

^b^Significant differences (*P*<.05) between high and low SAP detected using unpaired two-samples *t* test.

Results presented in Table 1 show that high SAP spent less time at leisure and food places and more time at home and out of town during both weekdays and weekends. However, during weekends, high SAP tended to spend more time at education places (around 148 minute) compared with low SAP (around 102 minute). They also appeared to spend less time at other houses (a difference of 48 minute), perhaps because they were less comfortable engaging in social interactions that may happen at friends’ houses or simply had fewer opportunities (invitations) for these interactions.

#### Distribution of Visits

**Figure 5:**
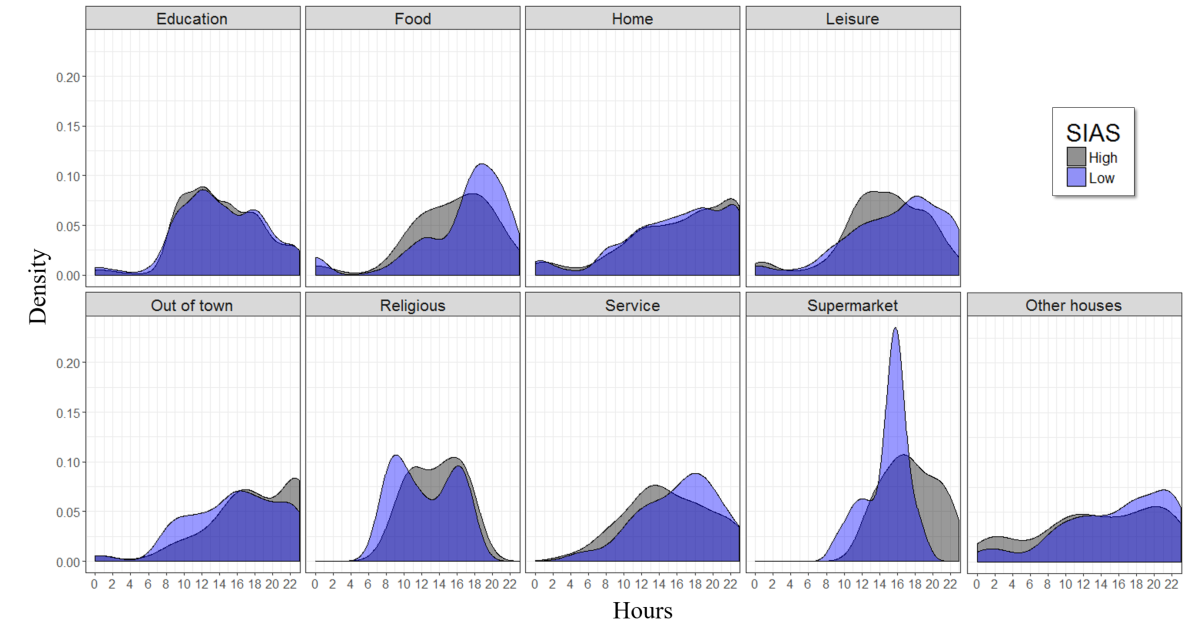
The distribution of location visits over the day for both high (grey) and low (blue) socially anxious participants. The Epanechnikov kernel function was used to compute the density estimates presented in this figure. SIAS: Social Interaction Anxiety Scale.

To better understand the relationship between students’ daily routines and their social anxiety, we analyzed the distribution of location visits over the day for both high and low SAP. Figure 5 illustrates the difference in the distribution of location visits between the two populations. Note that this figure analyzes the time of visits (the time that a participant visited a specific location) without considering the duration of visits because cumulative staying time has already been studied above.

The results show a difference in the pattern of visits to food places, supermarkets, others’ houses, and leisure places. Low SAP appeared to prefer going to friends’ houses and food and leisure places during evenings (after 4 pm) more than high SAP. On the other hand, high SAP preferred to stay at home or go to the supermarket during that time period. This suggests that there may be a difference in how students plan their daily activities based on how socially anxious they are. Understanding these patterns may help clinicians identify when a person is starting to withdraw more socially and both plan and easily monitor specific social activity targets in treatment.

For instance, it is common during cognitive behavioral therapy for social anxiety disorder to have clients plan novel social activities, both to have more opportunities to receive reinforcement from the environment and reduce withdrawal and to engage in previously avoided activities to learn that the social anxiety can be tolerated (these “exposures” are designed to reduce social avoidance and provide new learning opportunities). Having clients identify good opportunities for these social activities and monitoring what they did over the past week can be challenging; thus, this real-time monitoring could help with treatment planning and assessing progress in ecologically valid ways in real time.

#### Location Entropy

We calculated the correlation between location entropy and the SIAS score during weekdays and weekends. Results showed that the diversity of places visited on both weekdays and weekends was negatively correlated with the SIAS score (*r*=−0.64, *P*=.001 for weekdays and *r*=−0.57, *P*=.001 for weekends), which suggests that socially anxious students visit fewer *different* places and have a narrower range of activities. This finding is consistent with a previous work [7] concluding that location entropy is strongly related to feelings of sadness and stress among students. Importantly, tracking the range of places a person with social anxiety visits could be a very useful marker of treatment progress because increasing the range may indicate an expanded repertoire of social skills and engagement, given different activities require different types of social interactions (eg, attending a party at a house is quite different from having dinner on a date with one person). It also likely indicates a willingness to try new social activities.

#### Frequency of Transitions

Next, we investigated whether transition frequency between each different location correlated with SIAS scores. Table 2 shows the results of the Pearson correlation (with *P* values) between transition frequencies and SIAS scores. The total number of distinct transitions was 152. In Table 2, we have presented only transitions having a correlation coefficient higher than 0.3 and *P* value <.05. Results show that some transitions correlated positively with SIAS scores, such as going from education to supermarket, while other transitions correlated negatively with SIAS scores, such as going from one leisure place to another leisure place. These data provide convergent support for the earlier findings, suggesting that socially anxious individuals are less likely to do leisure activities later in the day (thus, do not transition from one leisure activity to another) and more likely to do more typically solitary activities like grocery shopping.

Studying transition periods may be useful clinically to the extent they represent potential instances of approach-avoidance conflict in socially anxious individuals [4]. A hallmark feature of social anxiety is avoidance of settings in which social scrutiny is likely to occur [28]. Therefore, individuals may be less likely to transition from socially secure locations to insecure locations and more likely to transition from socially insecure locations to secure locations. In addition, the frequency of transitions might be a meaningful metric, such that more transitions are made on days with a higher anxiety level. Studying transitions could, thus, ultimately allow for greater insight into decision making about approach and avoidance behaviors in the real world.

### Predicting Social Anxiety

Using the extracted mobility features, we investigated whether the mobility features could predict students’ SIAS scores. We studied the results of (1) the classification task that classified participants as low or high socially anxious using SIAS score=34 as a cutoff and (2) the regression task by predicting the actual SIAS score of a participant (between 0 and 80).

**Table 2:** Pearson correlation (with *P* values) between each participant’s Social Interaction Anxiety Scale score and his or her transition frequency from one location to another. Only transitions having a correlation coefficient > 0.2 and *P*<.01 have been presented.

Transitions	Observations	Pearson coefficient	*P* value
Out of town ⟶ Religious	45	0.337	0.011
Supermarket ⟶ Education	46	0.339	0.010
Education ⟶ Supermarket	133	0.223	0.004
Leisure ⟶ Other houses	112	−0.219	0.010
Out of town ⟶ Leisure	35	−0.282	0.050
Service ⟶ Leisure	45	−0.291	0.026
Leisure ⟶ Leisure	26	−0.495	0.005

For each day, we extracted 220 mobility features as follows: (1) cumulative staying time at each different location and during each different time window: 8 am-4 pm, 4 pm-12 am, and 12 am-8 am; (2) the distribution of visits over time (time series of locations visited during that day); (3) location entropy; (4) the frequency of each different transition; (5) the type of day (weekday or weekend); and (6) the day of the week (eg, Monday, Tuesday). Note that we did not include the actual date (ie, MM/DD/YYYY) to avoid overfitting because we were testing our approach over a short study period. In long-term studies, this feature can be added to track special events such as national holidays. Mobility patterns during these special events may be a predictor of social avoidance and social anxiety.

Theoretically, daily mobility features would all be included in the same model as predictors of trait social anxiety. However, in practice, this is not feasible because of the large number of dimensions for a small number of participants. In other words, for each day, each different place, each transition type, and each time window (morning, evening, etc) would be a different feature. Thus, the total number of features will increase with the number of days in the study. This phenomenon is known as *the curse of dimensionality* where the volume of the space increases so fast that the available data become sparse [29]. There are two traditional solutions to tackle this problem. The first is to aggregate the features on daily basis, which means, instead of having more than 3000 features for 228 participants, we determine only 220 aggregated features (average time spent at home, at university, etc during the study period). Thus, 220 is the number of distinct mobility features that we may have in a given day. We utilized this approach as one of our baseline measures (*BM*_1_). The second solution is to apply a dimensionality reduction technique such as principal component analysis (PCA) or autoencoders to select the most important features. We also applied this method as one of our baseline measure comparisons (*BM*_2_), reducing our feature space to between 50 and 200.

**Figure 6:**
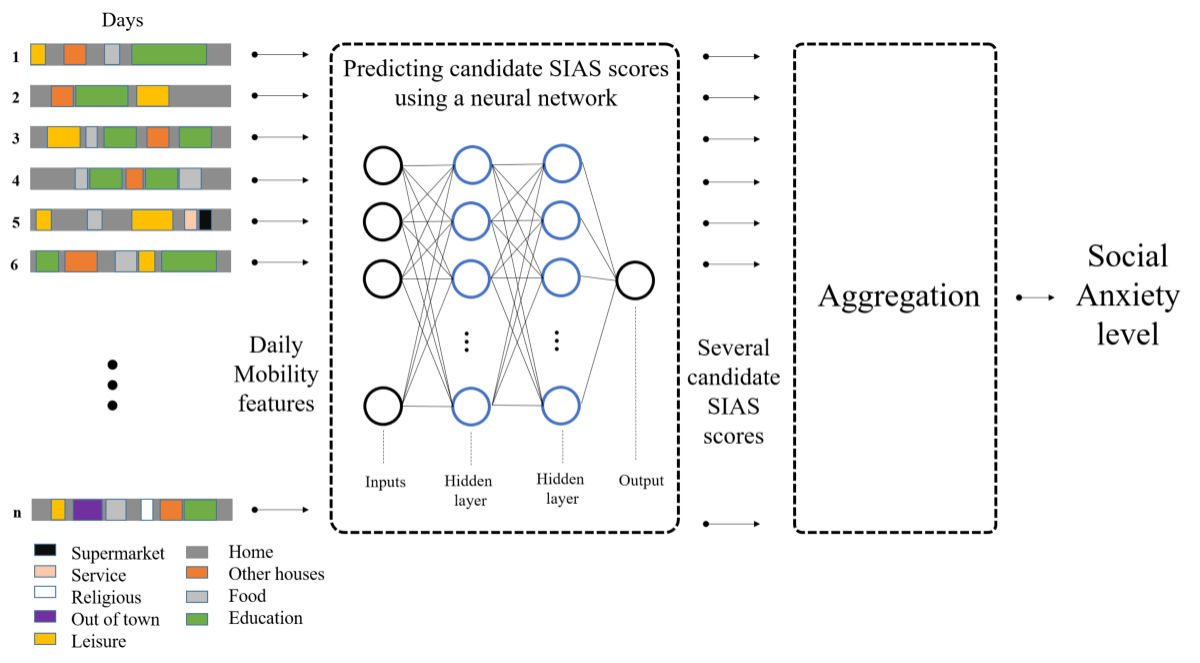
The architecture of the proposed prediction method. Daily mobility features are first extracted and then used to predict multiple candidate Social Interaction Anxiety Scale (SIAS) scores using a neural network. A global SIAS score is assigned to a participant by aggregating the predicted candidate SIAS scores.

Finally, we proposed a new method to predict a daily SIAS score, which uses a neural network with all 220 mobility features and compare it to the two traditional solutions (*BM*_1_ and *BM*_2_). Figure 6 describes the design of our method. We started by predicting candidate SIAS scores for each participant for each day of the study (ie, if a participant had 15 days, we predicted 15 candidate SIAS scores); then, in the second layer, a global predicted SIAS score was calculated by aggregating the daily candidate SIAS scores. For the regression task, the aggregation function used a 7% trimmed mean of the predicted daily SIAS scores; the trimmed mean helps eliminate the influence of predictions on the tails that may unfairly affect the traditional mean. However, for the classification task, the dominant class was chosen. If there was no dominant class (number of days predicted as low and high were the same), the aggregation function chose one class randomly (See aggregation in Figure 6).

Our method has the following advantages:

- Predicting on daily basis allows to capture low-level mobility features that may improve the performance of our predictor (eg, time spent at some locations during the evening of Fridays) without facing the curse of dimensionality.
- After predicting a candidate SIAS score for a given day, all raw GPS data and mobility features for that day can be deleted. Consequently, such methods can operate efficiently on mobile phones, which are known for their limited storage capacity.
- The proposed model operates incrementally and is independent of the number of days. This means it can predict social anxiety levels from the first days of the study, and it can predict SIAS scores for participants having unbalanced data (fewer number of days than expected). This is not possible with other baseline methods because they require all participants to have the same number of features (dependent on number of days) to be able to train and predict social anxiety.
- By having several candidate SIAS scores per participant, we are able to deal with days that might be outliers (ie, mobility patterns significantly different). Our method is designed to remove noise (using trimmed mean) that may be generated by unusual behaviors that may bias the data. For instance, one participant may behave as low socially anxious for only one day, while his or her behavior during other days will resemble a high SAP. This outlier will be automatically removed by our model.

The extracted features are used to train a neural network. We used a multilayer perception (MLP) [30] trained using a back propagation algorithm. It uses hyperbolic tangent activation function and contains 2 hidden layers with 100 nodes each (we did not notice a better performance by adding more nodes and hidden layers). Neural networks are popular models that have shown great promise in many tasks such as sentiment classification, image captioning, and natural language processing. We chose MLP because of its ability to detect nonlinear relationships between inputs and outputs; thus, it can detect hidden patterns between mobility and SIAS that we could not find in our linear analysis presented earlier (ie, correlations).

We compared our prediction method with two baseline models *BM*_1_ and *BM*_2_. *BM*_1_ is a nonincremental method that predicts the SIAS score on biweekly basis, ie, it waits until the end of the study to predict social anxiety levels. *BM*_1_ uses the same mobility features as our method but aggregated on the whole study period. For instance, rather than computing cumulative staying time for each location per day, *BM*_1_ computes the average daily cumulative staying time per location for the entire study period; it also does the same for the other features: entropy of location, frequency of transitions, and average cumulative staying time during weekdays versus weekends. We also compared our method to *BM*_2_ that uses PCA to reduce feature space from 3000 to 200 (less than the number of participants, which were 228). Note that we did not notice any improvement when we reduced feature space dimension to 50, 100, or 150. Both *BM*_1_ and *BM*_2_ use an MLP with a structure similar to our method (ie, 2 hidden layers, 100 nodes each).

Table 3 illustrates the overall performance of our method compared with that of *BM*_1_ and *BM*_2_ for both classification and regression tasks. The models were evaluated using leave-one participant-out cross-validation (LOOCV) and 10-fold cross-validation (FCV), where each validation fold contained 12 random high SAP and 12 random low SAP; the overall number of days in each fold was around 300 days. Table 3 presents three evaluation metrics averaged across folds: (1) root-mean-square error (RMSE) for the regression task and (2) accuracy and (3) F-1 measure for the classification task.

**Table 3:** Performance of our prediction method (OM) compared with that of two baseline methods, *BM*_1_ and *BM*_2_ for both classification and regression tasks. We used two evaluation methods: leave-one participant-out cross-validation (LOOCV) and 10-fold cross-validation (FCV); and three evaluation metrics: root-mean-square error (RMSE) for the regression task and accuracy and F-1 for the classification task.

Methods	LOOCV			FCV			
	RMSE	Accuracy	F-1		RMSE	Accuracy	F-1
	Mean (SD)	Mean (SD)	Mean (SD)		Mean (SD)	Mean (SD)	Mean (SD)
OM	7.06 (2.01)	0.85 (0.04)	0.87 (0.04)		7.7 (2.95)	0.81 (0.03)	0.85 (0.04)
*BM*_1_	11.87 (4.23)	0.69 (0.06)	0.72 (0.04)		12.23 (4.71)	0.65 (0.04)	0.69 (0.04)
*BM*_2_	10.95 (4.92)	0.72 (0.04)	0.75 (0.04)		11.31 (4.55)	0.71 (0.04)	0.69 (0.03)

**Figure 7:**
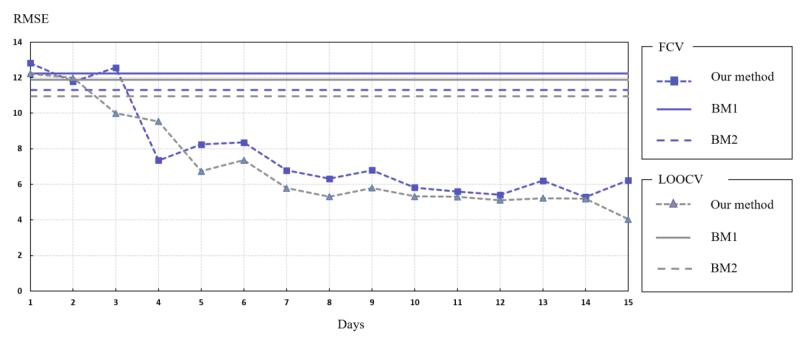
The performance of models over the course of the study. FCV: 10-fold cross-validation, LOOCV: leave-one participant-out cross-validation, RMSE: root-mean-square error.

Results show that our methods outperformed and for both classification and regression tasks and for both types of validation. For instance, for LOOCV, our method showed an accuracy of 85%, F-1 measure of 87%, and a RMSE of 7.06 compared with *BM*_1_, where we recorded an accuracy of 69%, F-1 measure of 72%, and a RMSE of 11.87, and *BM*_2_, where we recorded an accuracy of 71%, F-1 measure of 75%, and a RMSE of 10.93. The promising results of our hierarchical prediction method are justified by the fact that extracting and using daily features helps learn the different hidden patterns between mobility and SIAS that we presented above (eg, mobility in weekdays vs weekends, cumulative staying time at each different location during mornings vs evenings, and transition frequencies) and this while taking into account the day of the week; for instance, activities during Friday evenings (when students tended to engage in leisure activities) can be a predictor of social isolation. *BM*_1_ and *BM*_2_ failed to capture these low-level patterns that may play a role in predicting social anxiety, mainly because when aggregating on the study period basis or reducing feature space dimension, the model loses some information such as mobility during a specific time epoch (eg, morning), on a specific *type* of day (eg, Sunday).

Our prediction method operates incrementally, which means that it can predict an SIAS score starting from the first day of the study and update the estimated SIAS on each subsequent day of the study. Figure 7 depicts the average RMSE of all participants over the course of the study. The RMSE improved converging at about 7 study days. We hypothesized that this was because 1 week of data was necessary to observe behavioral patterns.

Even though our method provided superior results compared with baseline methods, it still had an error of 7.06 on an 80-point scale. One hypothesis is that this may be justified by the limitations of SIAS scores that may suffer from self-report biases [31]. Another limitation was the short study length that may not be enough to characterize the rhythms indicative of social anxiety.

## Discussion

### Principal Findings

In this paper, we demonstrated the feasibility of assessing college students’ social anxiety through GPS-based localization. Findings from this study suggest that it is possible to use passively sensed location data from mobile phones to predict social anxiety levels. We integrated semantic labels of locations, such as locations of leisure, into our prediction models. This provided a more nuanced understanding of the behavioral patterns of socially anxious individuals. For instance, consistent with the existing theory and psychological research, we found that socially anxious students tended to avoid locations that contained a threat of social scrutiny. High SAP infrequently visited food places and engaged in less leisure activities during evenings and weekends. Exploiting the temporal richness of passively sensed location data also revealed that socially anxious students spent more time at home after school between 4 pm and 12 am. This study also extended the prior work by presenting a predictive model based on neural networks. The results of our prediction method suggest that social anxiety can be efficiently predicted by mobility features. Importantly, our findings are based on objective behavioral data gathered from people’s daily lives and, therefore, avoid recall biases associated with self-reports of behavior.

While most research using mobile sensing has focused on depression, relatively little is known about the location features that might be indicative of a high social anxiety level. Social anxiety and depression share common symptoms and underlying factors, such as high negative emotionality, social withdrawal, and avoidance. However, there are also key differences. For example, depression is often marked by a slowing of movement (psychomotor retardation) and a general reduction of activity that is not found in social anxiety. Our results indicate that socially anxious individuals tend to spend time at home specifically after school, a time when additional social activities are less likely be mandatory (eg, classes are done). Thus, rather than demonstrating a general avoidance of all activities throughout a day (which could indicate a general lack of motivation or energy), this finding sheds light on the temporal nuances of social anxiety that may not be found in depression and suggests a pattern more specific to social avoidance. There may also be key differences in the types of places that are avoided; while some individuals with high depression may avoid all locations that require high energy (eg, going to a gym), those with high social anxiety levels may focus on avoiding places and times that pose the greatest risk of social evaluation (eg, places of leisure during peak hours, which fits our results). Our findings are among the first to examine mobility features of social anxiety using fine-grained GPS data from people’s daily lives. Future work leveraging mobile sensors has the potential to test and improve on existing psychological models. For example, our findings suggest that contextual factors such as location type and time of day may have an important impact on how people with social anxiety choose to isolate themselves.

### Limitations

Although our findings reveal mobility features associated with social anxiety in everyday life, it is important to stress that these findings are preliminary. First, our findings are based on an unselected sample of undergraduate students. While a homogeneous sample increases the internal validity of our findings, using university students could limit the generalizability of our results to nonstudent populations whose daily life experiences and activities are different [32]. Furthermore, while there was a good representation of high socially anxious individuals in our sample who met clinical cutoffs, a nonclinical sample of undergraduate students was used. Future research should test whether the mobility features identified in this study can be generalized to a clinically diagnosed population. Second, due to the correlational nature of the data, no causal claims between social anxiety symptoms and mobility features could be made. For example, we cannot exclude the possibility that other factors outside of social anxiety influenced our findings, such as alcohol use, which is a common way of coping with distress. We also did not correct for the possible effects of multiple comparisons, given our focus of exploring mobility features that may be candidates for future research. We hope the current work will be a launching point for subsequent researchers interested in using passively sensed data for detecting social anxiety. Third, given limitations of statistical power, we were unable to examine the potential moderating roles of gender and race, which should be more closely examined in future work. Finally, one issue that future work should address is the issue of multiple comparisons, which can inflate the chance of accidental discoveries. This usually arises when the same statistical tests are repeatedly computed from the same set of observations in a hypothesis-driven research [33]. Fortunately, there are several ways of addressing this issue, many of which require researchers to set a more stringent significance threshold [34,35]. For example, while many of the transition correlations presented in the current study remained significant at *P*<.01, it is important to note that we did not have *a priori* hypotheses regarding what specific types of location pairs would be most relevant. Thus, future work should always include the direction and magnitude of effect sizes. Finally, research also suggests that different types of analyses are less prone to bias from multiple comparisons, such as those based on a hierarchical Bayesian framework [36].

### Conclusion

In spite of the limitations, the ability to use fine-grained GPS data to detect behavioral patterns associated with social anxiety has many important implications. It opens up the possibility that health care professionals can identify and monitor those in need of help, but struggle with the prospect of initiating contact with others. With the increasing prevalence of social anxiety disorder and other mental health concerns, novel techniques for assessing psychological distress have become increasingly important. By leveraging the ubiquity of mobile phones and their increasingly powerful sensors, researchers and clinicians might finally be able to overcome many of the traditional obstacles to providing care.

## Abbreviations

FCV: 10-fold cross-validation
GPS: Global Positioning System
LOOCV: leave-one participant-out cross-validation
MLP: multilayer perception
OSM: open street map
RMSE: root-mean-square error
SAP: socially anxious participant
SIAS: Social Interaction Anxiety Scale
